# Supplementary material for: Association between wasting and inadequate breastfeeding practices among infants under six months in SNNPR and Somali regions of Ethiopia: A multilevel cross-sectional study
Source: PLoS One. 2025 Feb 7;20(2):e0318323. doi: 10.1371/journal.pone.0318323 (PMC11805366; doi:10.1371/journal.pone.0318323)
Supplement: S1 Table — (DOCX) [file pone.0318323.s002.docx]

**S1 Table. Explanatory variables categorization and coding.**

| Variables | Category |
| --- | --- |
| Age of the mother (years) | 1. 15-19 2. 20-24 3. 25-29 4. 30-34 5. >35 |
| Mother education | 1. No formal education 2. Primary education 3. Secondary and above |
| Source of drinking water | 1. Improved source (Piped into dwelling, plot, yard) 2. Other improved sources (Protected well, spring, rainwater) 3. Non-improved source (pond, lake, dam, river, open well, spring) |
| Toilet facility | 1. Non-improved facility (Pit latrine/traditional pit toilet) 2. Improved facility (Ventilated improved pit latrine (VIP) 3. No facility/bush/field |
| Wealth index | 1. Poor 2. Poorer 3. Middle 4. Rich 5. Richer |
| Maternal Height (m) | 1. Short 2. Medium 3. Tall |
| Number of pregnancies  Including the index child | 1. 1-2 2. 3-4 3. ≥5 |
| Number of living children, Including the index child | 1. 1-2 2. 3-4 3. ≥5 |
| First ANC visit for the index child (month) | 1. <3 month 2. 3-6 month 3. >6 month |
| Frequency of ANC visits for the index child | 1. 1-3 2. ≥4 |
| Place of delivery of the index child | 1. Home 2. Health facility |
| Mode of delivery of the index child | 1. Vaginal delivery 2. Caesarian section |
| Child sex | 1. Male 2. Female |
| Child age  (completed month) | 1. 0 2. 1 3. 2 4. 3 5. 4 6. 5 |
| Frequency of breastfeeding, yesterday during the day and night | 1. < 8 times 2. > 8 times 3. Not breastfeed |
| Perceived birth size of the index child | 1. Very small 2. Average 3. Very big |
