# Supplementary material for: Association between wasting and inadequate breastfeeding practices among infants under six months in SNNPR and Somali regions of Ethiopia: A multilevel cross-sectional study
Source: PLoS One. 2025 Feb 7;20(2):e0318323. doi: 10.1371/journal.pone.0318323 (PMC11805366; doi:10.1371/journal.pone.0318323)
Supplement: S2 Table — (DOCX) [file pone.0318323.s003.docx]

**S2 Table. Variables used for constructing the wealth index.**

| Variables | | Category |
| --- | --- | --- |
| 1 | Main floor material not finished, finished (tile, ceramic) | 1. No 2. Yes |
| 2 | Main roof material (no roof and finished roof) | 1. No 2. Yes |
| 3 | Main exterior wall material (no walls and finished wall) | 1. No 2. Yes |
| 4 | In your household, does anyone own Metal cooking pots/pans | 1. No 2. Yes |
| 5 | In your household, does anyone own Bucket | 1. No 2. Yes |
| 6 | In your household, does anyone own Plates/bowls | 1. No 2. Yes |
| 7 | In your household, does anyone own Cup/mug | 1. No 2. Yes |
| 8 | In your household, does anyone own Radio | 1. No 2. Yes |
| 9 | In your household, does anyone own TV | 1. No 2. Yes |
| 10 | In your household, does anyone own Mobile telephone | 1. No 2. Yes |
| 11 | In your household, does anyone own Improved charcoal wood stove | 1. No 2. Yes |
| 12 | In your household, does anyone own Kerosene stove/wood burner | 1. No 2. Yes |
| 13 | Wooden bed | 1. No 2. Yes |
| 14 | Jewelry (gold, silver, wristwatch) | 1. No 2. Yes |
| 15 | Modern chair | 1. No 2. Yes |
| 16 | Modern table | 1. No 2. Yes |
| 17 | Cow/oxen | 1. No 2. Yes |
| 18 | Goats/sheep | 1. No 2. Yes |
| 19 | Chicken/duck | 1. No 2. Yes |
| 20 | Donkey/horse/mule | 1. No 2. Yes |
| 21 | Camel | 1. No 2. Yes |
