# Supplementary figures and images for: Association between wasting and inadequate breastfeeding practices among infants under six months in SNNPR and Somali regions of Ethiopia: A multilevel cross-sectional study
Source: PLoS One. 2025 Feb 7;20(2):e0318323. doi: 10.1371/journal.pone.0318323 (PMC11805366; doi:10.1371/journal.pone.0318323)

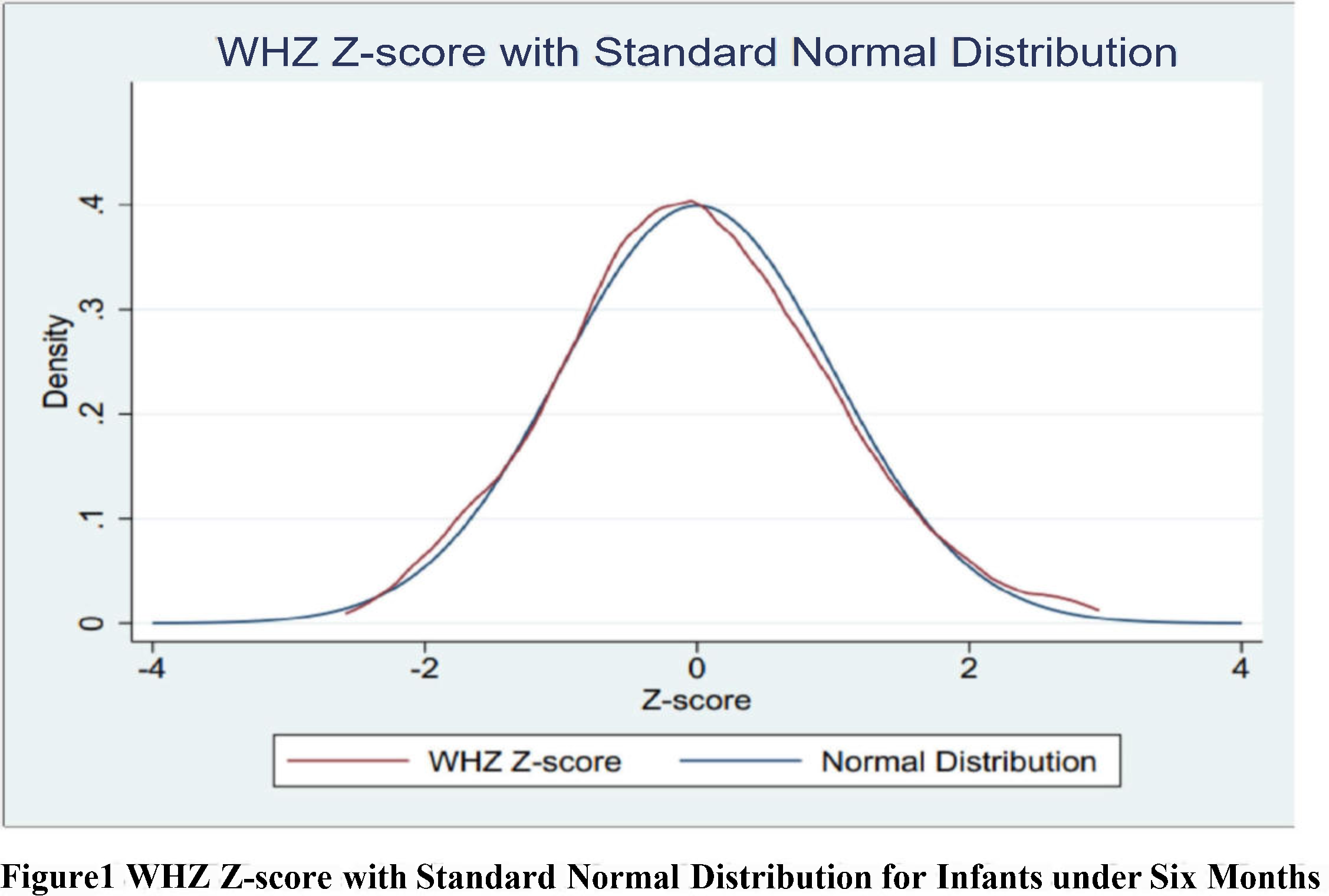

Supplement: S1 Fig — (TIF) [file pone.0318323.s004.tif]
