# Supplementary material for: Association between wasting and inadequate breastfeeding practices among infants under six months in SNNPR and Somali regions of Ethiopia: A multilevel cross-sectional study
Source: PLoS One. 2025 Feb 7;20(2):e0318323. doi: 10.1371/journal.pone.0318323 (PMC11805366; doi:10.1371/journal.pone.0318323)
Supplement: Attachment — Submitted filename: Response to Reviewers.docx [file pone.0318323.s005.docx]

**Summary of Response to Reviewers Comment**

Regarding the study period, data collection for this study took place in Ethiopia during the Ethiopian calendar year 2013. The Ethiopian calendar system is approximately seven to eight years behind the Gregorian calendar. This difference arises due to an alternate calculation of the birth year of Jesus Christ used in the Ethiopian calendar. The Ethiopian year begins on Meskerem 1, which typically falls on September 11 in the Gregorian calendar (or September 12 in a leap year). The initial portion of Ethiopian year 2013 (from Meskerem, which starts in September, through December) corresponds to September–December 2020 in the Gregorian calendar. The latter part of Ethiopian year 2013 (from January through August) aligns with January–August 2021 in the Gregorian calendar. Consequently, while the dataset indicates an Ethiopian calendar year of 2013, it actually encompasses a timeframe that includes late 2020 and most of 2021 in the Gregorian system.

The use of a p-value threshold of less than 0.25 for variable selection in multivariable analysis was chosen to ensure potentially relevant predictors are not excluded prematurely. While the standard p-value of 0.05 is often used to determine statistical significance, a more inclusive threshold, such as 0.25, is applied during initial screening in bivariable analysis to capture variables that may not show strong individual associations with the outcome but could be influential when considered together with other predictors (Bursac et al., 2008). This will minimize the risk of excluding important variables that could improve the model's predictive ability and robustness in multivariable contexts. This statement is also included in the revised manuscrpt on the data analysis part. *“All the variables with a p-value of less than 0.25 were used during initial screening in the bivariable analysis to capture variables that may not show strong individual associations with the outcome but could be influential when considered together with other predictors. (Zoran B., et al 2008).*

Additionally, we included the prevalence of exclusive breastfeeding in the study area, in the discussion part. “*According to the 2019 Ethiopia Demographic and Health Survey (EDHS), the prevalence of exclusive breastfeeding for infants under 6 months in the SNNPR, 58%. In contrast, the rate in the Somali Region is lower, at around 25%.” (EDHS, 2019)*

**Reviewer Comments**

1. Take seriously the following major concerns regarding the data periods claimed. The dates of birth in the dataset are all in 2013 but the study claims to have been carried out in 2021. Justify this discrepancy.

**Response: “This concern has been addressed in the summary section provided above.”**

2. Along with global statistics, mention the statistics of exclusively breastfeeding in the study area.

**Corrected: Yes, “This concern has been addressed in the summary section provided above.”**

3. From the Study design and setting, delete the sentence “The study aimed to strengthen nutrition interventions integrated into existing antenatal care services and to improve maternal nutrition practices.

**Corrected: Yes, *this sentence is deleted from the Study design and setting. “The study aimed to strengthen nutrition interventions integrated into existing antenatal care services and to improve maternal nutrition practices.”***

4. The authors write “All the variables with a p-value of less than 0.25 in bivariable analysis were used for multivariable analysis.” What is the reason for using a p-value of less than 0.25? Justify your choice and add a reference(s). Is it “multivariable” or “multivariable” check it carefully. There is a huge difference between them.

**Response: Yes, “This concern has been addressed in the summary section provided above.” *The revised statement is also included in the revised manuscrpt on the data analysis part.***

*Thank you for your concern, the term "multivariable" refers to models involving multiple predictors for a single outcome, whereas "multivariate" is used for models with multiple outcomes. In this analysis, "multivariable" is appropriate since we are focusing on multiple independent variables predicting one outcome.*

5. It is recommended to show the p-values in bivariate analysis.

**Corrected: Yes,** ***p-values in bivariate analysis is included.***

6. Limitations of the study should be a separate section.

**Corrected: Yes**, ***the limitation is written in the separate section.***

**Reviewer #1**

Reviewer #1: Thank you for the opportunity to review your work. The underlying approach is sound and many of the comments below are about making the work easier to read, or more precise. However, there is a major concern re. data periods claimed. The dates of birth in the dataset are all in 2013 but the study claims to have been carried out in 2021. It is for this reason I have recommended major revision.

MAJOR POINTS
INTRO
Authors take a long time to state this aim of this study. Suggest adding one or two lines to the end of the first paragraph that states the aim of this work was to look at the association between breastfeeding practices and wasting in infants aged under six months in the South of Ethiopia.

**Corrected:** **Yes, corrected in the manuscript as follows, *“the aim of this study was to examine the association between breastfeeding practices and wasting in infants under six months of age in the SNNPR and Somali regions of Ethiopia.”***

METHODS
Data claims to be from 2021 but the included dataset has all of the children's date of birth in 2013.

**Response: “This concern has been addressed in the summary section provided above.”**

RESULTS
Authors discuss LAZ and WAZ in Intro/Results but never refer to it in the manuscript again. Remove any mention of these variables if they are not discussed in detail outside of Table 3.

**Corrected:** **Yes, *we removed LAZ and WAZ from the Introduction and Results sections.***

DISCUSSION
There is no discussion of null or negative findings. For example, a wealth index is included but is not addressed in the discussion. Similarly, the number of pregnancies and births should be discussed at least briefly as to why they do or not affect the results.
**Corrected:** **Yes, *On the discussion part the null or negative findings were discussed. This statement is included in the discussion part as follows.* “While the wealth index showed a significant association with infant wasting in the bivariate analysis, this association did not hold in the multivariable analysis when other factors such as breastfeeding practices and maternal education are considered. One possible explanation is that wealth may indirectly influence wasting by affecting these more proximal factors, such as the mother's education level and access to health information, which in turn influence infant feeding practices (Hammond YA, et al., 2024). When these factors are controlled for in the multivariable analysis, the direct association between wealth and wasting diminishes. Additionally, wealth may capture general household resources but may not fully reflect specific nutritional or healthcare practices crucial for preventing infant wasting. Thus, while wealthier households might have better resources, the actual practices around infant feeding and care are key determinants of infant nutrition are more directly relevant to wasting outcomes** **(Lijalem MT et al 2021). This analysis also explored whether the number of pregnancies and births (parity) was associated with infant wasting. Although higher parity is often thought to affect child health outcomes due to resource allocation or maternal experience, our findings did not show a significant association between the number of pregnancies, parity and wasting in the multivariable analysis.**

**One possible reason for this non-significance is that factors more directly tied to the immediate care and feeding of the infant, such as exclusive breastfeeding and early initiation of breastfeeding, may be stronger determinants of wasting. The mother’s experience with multiple births may increase her general caregiving knowledge but does not necessarily ensure the specific practices that prevent wasting. Furthermore, higher parity can sometimes strain household resources and maternal time, but these factors may not directly impact infant nutritional status if the household or mother is already practicing adequate breastfeeding practices. (Renata O.N, et al., 2021).**

MINOR POINTS
Abstract
Line 26 - Suggest removing "inadequate".

**Corrected: Yes,** ***"inadequate" has been removed.***

Line 27 - South East, not Southeast.

**Corrected: Yes,** ***to ensure clarity, I revised the text to explicitly mention both the SNNPR and Somali Regions in the manuscript.***

Line 27 - SNNPR is in the South West of the country. Describing the work as being set only in South East is incorrect.

**Corrected: Yes, corrected as discussed above.**
Introduction
Line 75 - Capitalisation of World Health Organization

**Corrected:** **Yes, corrected to “World Health Organization”.**

Lines 80-84 - This paragraph does not add to the value of the introduction. Consider removing it because the introduction is very long already.

**Corrected: Yes, *removed the paragraph written in Lines 80-84.***

Line 87 - "The prevalence of wasting..." should not be a new sentence but should carry on from the first sentence in the paragraph.

**Corrected:** **Yes, corrected by combining the sentences as follows.** “***Despite the implementation of various strategies and programs, including the National Nutrition Program (NNP), the prevalence of wasting among children under five remains high in Ethiopia.”***
Line 92 - This paragraph starts by mentioned underlying factors but goes on to discuss problems with measurement methodologies. Consider revising first sentence. Line 102 onwards seems to discuss underlying factors but appears to be a new paragraph.

**Corrected:** **Yes, *the paragraph stated in Line 92 is removed and discussed the underlying factors in Line 110.***
Line 99 - WLZ initialism was defined earlier but "Weight-for-Length Z-score" is written out in full here.
**Corrected:** **Yes,** "**Weight-for-Length Z-score" *changed to “WLZ”.***

Methods
Line 124 and elsewhere - It is unclear what the "end-line" survey is.

**Response:** **The study had a base-line and end-line household survey on a large feasibility and impact study of maternal nutrition interventions. For the current study I used the end-line household survey data.**

Line 130 - Data period claims to be 2021 but dates of births in dataset are 2013.

**Response: “This concern has been addressed in the summary section provided above.”**

Line 149 - Were all children singleton births? Multiple births can have lower birth weight which may be relevant to growth comparisons in the early months of life.

**Response. "Thank you for raising this important point. While we acknowledge that multiple births can influence birth weight and subsequent growth comparisons, but this was not flagged as a significant issue during data collection, and the data collectors did not report encountering such cases. Additionally, our dataset does not include specific information on whether children were singleton or multiple births, which limits our ability to address this factor directly in the analysis. We appreciate your understanding and will consider this variable in future studies."**

Line 154 - It is unclear what is meant by "enumerator training and field practice".

**Response:** **In this study, we used secondary data, so the term 'enumerator training and field practice' refers to the preparation and practices undertaken by the original data collection team. In the primary data collection phase, enumerators were trained to ensure standardized and accurate data collection, and they conducted field practice to familiarize themselves with the data collection tools and procedures before gathering data in real settings. This training and practice aimed to improve data reliability and consistency.**

Line 171 - Is zcore06 function from a third-party package? If so, please include a citation.

**Corrected: Yes,** ***the zscore06 function is part of a user-written Stata package, specifically designed for calculating child growth z-scores using WHO standards. We used Stata version 17, and we have included a citation in the manuscript. (Leroy JL. (2011)***

Line 175 - LAZ and WAZ do not appear to be mentioned anywhere else in the article to please remove this line.

**Corrected:** **Yes, *LAZ and WAZ removed from the manuscript.***

Line 178 - Was mother height (included in the supplied dataset) used in any of the models? If so, was it the continuous variable or the categorical in the dataset?

**Response: Yes, it was included in the supplied dataset, but it was not used in any of the models, because the p-value is above 0.25 in the bivariate analysis.**

**The maternal height is categorized in to three by using a command, xtile mother-heigh = height1, nq (3)**

**Mother-heigh was labeled as 1 "short" 2 "medium" 3 "tall". It was included in the explanatory variable of the manuscript.**

Line 183 - Was the "child perceived birth size" variable used anywhere? If so, this should be recognized as a limitation since it is subject to recall bias and a subjective variable.

**Response: The child perceived birth size variable is described in numbers in Table 3. *But not included in any of the model because in the bivariate analysis the p-value is above 0.25.***

Line 191 - What is an "index child"? No definition given.

**Corrected: Yes, this definition is included in the explanatory variables. “*The index child is the specific child selected for the study during data collection, chosen from among the children available in the household.****”*

Line 192 - Use semi-colons to delineate between items in the list.

**Corrected: Yes,** ***semi-colons were used to delineate between items.***

Line 210 - The citation for the wealth index is a technical report but I couldn't find where it lists the consumer items. A more in-depth explanation of how the wealth index is calculated in this study is required. This may be a supplemental file that lists the "20 items" that the authors describe, and how they used these to divide in to quintiles.

**Corrected: Yes,** **twenty-one variables were used to develop the wealth index, and included as a supplemental file by listing all the twenty-one variables.**

To clarify the calculation steps for the wealth index, here is the procedure by using Stata commands:

1. **Conducting PCA and Checking Sampling Adequacy**
   First, we used Principal Component Analysis (PCA) to create a composite measure of wealth. To ensure the dataset was adequate for PCA, we checked the Kaiser-Meyer-Olkin (KMO) measure of sampling adequacy: estat kmo
2. **Extracting the First Component**
   We generated the first principal component from the PCA results as an asset score, which represents the wealth index score: predict comp1 rename comp1 asset_scor
3. **Creating Wealth Quintiles**
   Next, we divided the asset score into quintiles (five equal groups), with each group representing different wealth levels. This was done using the xtile command, which created the asset_inde variable with values ranging from 1 to 5: xtile asset_inde = asset_scor, nq(5)
4. **Labeling the Wealth Categories**
   Finally, we labeled each quintile to define wealth categories from "poor" to "richer." We used the recode and label define commands to assign descriptive labels to each group: recode asset_inde (1=1 "poor") (2=2 "poorer") (3=3 "middle") (4=4 "rich") (5=5 "richer"), gen(Wealth_index) label define asset_inde 1 "poor" 2 "poorer" 3 "middle" 4 "rich" 5 "richer", replace

Line 237 - Suggest adding "only", e.g. "Model I was adjusted for 238 socio-demographic variables ONLY, Model II was adjusted for maternal-related variables ONLY..."

**Corrected:** **Yes, corrected as follows, “Model I was adjusted for socio-demographic variables only, Model II was adjusted for maternal-related variables only, Model III was adjusted for infant-related variables only.”**

Methods general note - Explain that there may be some uncertainty around the child's day of birth so some ages were given to the nearest month, only. Explain how missing or ambiguous data were handled.

**Corrected: Yes,** **this statement is included in the data collection procedure part as follows. “*For the original study, missing or ambiguous data on birth dates were managed by verifying records, using parental recall based on significant events, and consulting healthcare workers to estimate infants' ages. When exact birth dates remained uncertain, ages were approximated to the nearest month to maintain consistency in analysis. These methods minimized the impact of missing or ambiguous data.”***

Line 241 - Please add a statement that Table 5 shows which variables were included in each of Models I-IV.

**Corrected:** **Yes, this statement was added at the end of line 241. “*Table 5 shows which variables were included in each of Models I-IV.”***
Line 241 - P values should not be conflated with confidence intervals. P values should not be mentioned here.

**Corrected: Yes,** ***The P value statement removed.***
Results
Line 274 - ResultS rather than Result

**Corrected: Yes, changed to *Results***

Line 276 - Inconsistent use of numbers and proportions across this paragraph. Sometimes numbers and percentages are presented, sometimes just percentages.

**Corrected: Yes, *corrected by writing only the percentage for all the prevalences.***

Line 276 - Most of these statisticss are presented in Table 1. This paragaph could be much shorter.

**Corrected: Yes, *the last sentence was removed.***
Line 280 - SNNPR should be redefined in Methods section or use "Southern". Just be consistent in which term you use.

**Corrected: Yes, corrected by writing SNNPR and Somali regions of Ethiopia throughout the manuscript.**

Line 283 - "Treat drinking water" - Treatment of drinking water is not mentioned in the variable definitions in the Methods section.

**Corrected:** **Yes, it was added in the explanatory variable as follows. *“Treatment of drinking water were coded as "no" and "yes".***

Line 293 - "Regarding the number of living children, 33.9% had more 294 than five children living with them, while 33.5% had one to two living children 295 and 38.6% of the mothers had more than five pregnancies." I found this sentence very difficult to follow. It made me wonder what is the importance of the number of pregnancies versus births, which is not discussed anywhere. Were pregnancies versus births a self-reported number, for example?

**Response:** **"Data on the number of pregnancies, births, and living children (including the index child) was collected as part of a comprehensive assessment of maternal reproductive history, which is known to impact maternal and child health outcomes, including nutritional status indicators like infant wasting. These figures were self-reported by the mothers during data collection.**

**Understanding the total number of pregnancies and births can reveal patterns of maternal health and reproductive history that influence infant health outcomes:**

**Pregnancies vs. Births: In this study, 38.6% of mothers reported five or more pregnancies, while 36.8% reported five or more births. A higher number of pregnancies relative to live births can indicate experiences with miscarriage, stillbirth, or other adverse reproductive events, which can impact maternal health and caregiving capacity, potentially affecting the nutritional status of surviving children.**

**By examining reproductive history alongside infant wasting status, we can better understand how maternal and family-level factors interact to influence child health outcomes. In households with more children, and especially in cases where multiple pregnancies did not result in live births, the increased burden on maternal resources may elevate the risk of undernutrition and wasting in early childhood."**

Line 305 - Sex percentages are different to the values presented in Table 3.

**Corrected**: **Yes, *the percentage of the sex are corrected.***

Line 321 - Why are there square brackets?

**Corrected: Yes, the square brackets removed.**
Line 322 - The prevalences (58 and 41.8) are much higher than the previous line (16.5%). Rewrite to clarify. Suggest that the prevalence is given for each sex, rather than the split by sex in the group of children in those that are wasted.

**Corrected:** **Yes, corrected by stating as follows “*The overall prevalence of wasting among infants was 16.5% (95% CI: 14.1, 19.2). Within this group, male infants had a higher prevalence, accounting for 58% of wasted cases, while female infants accounted for 41.8%”.***

Line 323 - Suggest to describe trends with age in the wasted and non-wasted groups rather than listing specific values. The values are all in Table 4 for the interested reader.

**Corrected: Yes, the trends with age in the wasted and non-wasted groups was described in the paper as follws. “*The proportion of wasted children varies with age, wasting is higher at 5 months, where 26.2% of wasted cases are observed. Wasting is lowest at 1 month (8.5%) and shows an increase at 2 months (20.6%), remaining relatively high from 2 to 5 months. This pattern suggests that the prevalence of wasting increases with age, especially from 2 months onward, reaching its highest point at 5 months. The proportion of non-wasted children fluctuates with age but generally shows a peak at 5 months, where 24.7% of non-wasted children are found. The percentage of non-wasted children is lowest at 0 months (9.3%) and then increases, with a notable rise at 2 months (20%) and 5 months (24.7%), suggesting a relatively higher proportion of non-wasting at these older ages*.”**

Line 326 - Similarly, make a short description of any trend with wasting and education level but let the reader look at Table 4 for exact figures.

**Corrected: Yes, the trends with maternal education in the wasted and non-wasted groups was described in the paper. “*A higher proportion of wasted children (66.7%) are from mothers with no formal education, indicating an association between lower maternal education and higher rates of child wasting. Among mothers with primary education, 31.2% of children are wasted, which is lower than those with no formal education. Only 2.1% of wasted children are from mothers with secondary or higher education, suggesting that higher maternal education is associated with a lower prevalence of child wasting.”***

Line 340-343 - Repetitious of methods section (Lines 245-247). Remove here or remove from methods section.

**Corrected: Yes, removed from the Line 340-343.**

Line 346 - P values described in Methods section but CI's are reported in results. Clarity should be made in Methods section to talk about CI's and what percentage CI is being used. Please also state 95% CI rather than just CI in the brackets.

**Corrected: Yes, in the method section this statement is added as follows. “*The prevalence of wasting was determined using proportions with 95% confidence intervals (CIs), providing a range within which the true population parameters are expected to lie with 95% confidence, adding insight into the precision and reliability of our estimates.”***

Line 344 - I do not believe that describing an APR of 0.62 as 38% lower is technically correct, but this may just be a semantics issue.

**Response. "Thank you for your observation. While we understand your concern, the description of an APR of 0.62 as 38% lower is based on standard interpretation, where a relative measure below 1 is expressed as a percentage reduction (1 - 0.62 = 0.38 or 38%). We acknowledge that this may come across as a matter of semantics, Therefore, we have retained this phrasing in the manuscript."**

Line 349 and 352 - Inconsistent levels of precision on the estimates. The estimate is rounded to 1.5 but CI given as 1.09, 2.07 for example. Stick to same number of sig fig (so report as 1.50 inc. trailing zero).

**Corrected: Yes, *corrected by adding the numbers, 1.5 to 1.50 for all the numbers including the numbers written in the Table 5.***

Line 352 - Suggest change to "Infants WHOM initiated breastfeeding MORE than one after birth..." to make it clear that these were children that experienced delayed first feeding.

**Corrected:** **Yes, corrected as follows “Infants whom initiated breastfeeding more than one hour of birth had a 52% higher prevalence of wasting compared with infants who started breastfeeding within one hour of birth.”**

Table 5 - Check levels of precision in all reported numbers. There are a couple of cases where a trailing zero is missing. For example, maternal age 25-29 column Model I = 1.9 (0.84-4.25) should be 1.90 and Pregnancies > 5 Final Model is 1.17(0.7-1.95) when it should be 1.17 (0.70-1.95).
Table 5 - CIs are reported with inconsistent formatting. Commas are used in the text but hypens in the Table. Please be consistent and use commas or hyphens throughout.

**Corrected:** **Yes, *the levels of precision in all reported numbers were corrected. And corrected by using only hyphen.***
Table 5 - Bold font OR asterisks should be used to indicate sig values, not both.

**Corrected:** **Yes, *corrected by using only asterisks.***

Table 5 - Footer - I don't know what the note about crude prevalence refers to in this table,

**Response: crude prevalence used to indicate the abbreviation used (CPR) specifically.**

Results general note - Did wealth index matter in terms of wasting prevalence?
**Corrected:** **Yes, *this was discussed in the discussion part in detail.***

Discussion
No comparison of the results with the most relevant study (citation #15, mentioned in introduction).
**Corrected:** **Yes, for infants under six months, the 2019 Ethiopian Demographic and Health Survey (EDHS) data and all the national surveys often aggregate data under broader age ranges, typically 0-59 months, rather than providing specific data on wasting for the 0-6 months age group alone. *“But the prevalence of wasting among children under five years, in the Somali region is, 21.4% According to the 2019 Ethiopian Demographic and Health Survey (EDHS). In the Southern Nations, Nationalities, and Peoples' Region (SNNPR) reported wasting at 6.3% for children under five years.” this sentence is included in the discussion part.***

Line 372 - Unnecessary square brackets about prevalence estimate.

**Corrected**: **Yes, *the square brackets removed.***

Line 377 - Nairobi COUNTY rather than country?

**Corrected: Yes, *changed Nairobi country to county.***

Lines 405-409 - Please report the prevalence ratios from the studies cited. This will allow the reader to see how these ratios compare in other contexts.

**Corrected: Yes, *it is included in the discussion part.***

Line 417 - Please add something to the end of this paragraph that essentially says "Our results therefore add to the evidence that breastfeeding is a vitally effective practice in the first months of a child's life and mothers should be encouraged and supported.." etc etc

**Corrected:** **Yes, *the sentence included as follows “Our study’s findings underscore that exclusive breastfeeding is far more than a feeding choice; it is a crucial, evidence-based intervention. These results add to the growing body of evidence affirming the essential role of exclusive breastfeeding in promoting healthy growth and reducing the risk of wasting in infants during their earliest months. Strengthening efforts to support exclusive breastfeeding is therefore vital in combating infant malnutrition. This reinforces the importance of breastfeeding as a powerful intervention for infant health, and mothers should be encouraged and well-supported to exclusively breastfeed as a core strategy for improving child health outcomes and reducing malnutrition.”***

Line 430 - Please add further critical discussion about what feeding within the first hour means. Does it relate to the child's in utero development such that a better-developed newborn is more able to initiate feeding? Or is it a cultural or health misinformation issue (in the same way colostrum discarding is)? The authors have explained the associations and consequences of early feeding well, but more detail about why an infant may or may not feed in the first hour is important to this discussion point.

**Corrected:** **Yes, included as follows “*This “critical window” within the first hour after birth is a crucial period when infants are most alert and ready to begin breastfeeding. Natural behaviors like rooting (the reflex to turn toward the breast) and suckling (the ability to latch and draw milk) are activated at birth, fostering a strong mother-infant bond and supporting successful early feeding (ACOG, 2023)***

***However, an infant’s readiness to breastfeed can be influenced by prenatal factors. Preterm infants or those with complications often have less developed reflexes and slower physiological responses, which can hinder effective latch and feeding. Additionally, conditions like low birth weight or birth asphyxia can reduce alertness and weaken reflexes, leading to delays in breastfeeding initiation (Aryal et al., 2022). Furthermore, cultural beliefs and health misinformation create further barriers, particularly in regions where traditional practices influence infant care. In some cultures, colostrum is mistakenly viewed as “dirty” or “unhealthy,” and caregivers may discard it, delaying the initiation of breastfeeding until what they perceive as the “clean milk” appears, typically a few days post-birth. Moreover, some mothers are advised to delay feeding to let the infant “rest” or because of concerns about pain or discomfort associated with breastfeeding. These cultural barriers are often compounded by healthcare misinformation and inadequate counseling during prenatal care. Without proper guidance on the benefits of early breastfeeding, mothers may delay initiation or avoid it altogether (ACOG, 2023). Thus, promoting early breastfeeding initiation requires both infant readiness and robust education and awareness to counteract these cultural beliefs and misinformation (Aryal et al., 2022***

Line 447 - It seems important to mention there may be cultural factors underpinning this sex difference. Or at least for the authors to mention if there is a lack of research on potential cultural factors. Pre-pubescent children are relatively similar between sexes.

**Corrected: Yes,** **included as follows** “***The higher vulnerability of male infants to wasting, compared to females, explained by the gender allocation of resources in many societies, particularly in feeding practices, caregiving, and healthcare access. In some cultural contexts, boys and girls receive different levels of care based on societal preferences for one gender over the other. For instance, some cultures prioritize feeding male infants with larger portions or more frequent feedings, which are thought to support their growth and future roles as providers or leaders (Ghosh, R., & Siddiqui, Z. (2022). In contrast, in cultures where girls are seen as more valuable for future reproductive roles or caregiving responsibilities, they may be provided with better nutrition during early childhood, which could shield them from wasting. In contrast, boys might face neglect or less attention, which can leave them more vulnerable to malnutrition and wasting (Raghunathan, K., Kannan, S., & Quisumbing, A. R. (2019).***

***Understanding these culturally rooted disparities is critical, as they suggest the need for research on how early caregiving practices and societal gender roles uniquely affect the growth and health of male and female infants. Such research would allow for targeted interventions addressing sex-specific vulnerabilities, ensuring that both boys and girls receive adequate care and nutritional support during the formative first six months of life (Bouis et al., 2019). Strengthening evidence-based policies to bridge these gender disparities in infant nutrition and health access is vital for reducing early-life malnutrition and promoting equal health opportunities for all infants.***

Line 453 - What would the implications be on your estimates of prevalence if you could account for morbidity? (Presumably children that experienced extreme wasting sadly died so would not be included, thereby making your estimate an underestimate?)

**Response: Indeed, if morbidity were accounted for in the analysis, it is likely that the estimates of prevalence would be somewhat underestimated. Specifically, infants who experience extreme wasting are at a higher risk of mortality, and those who die would not be included in the study sample, thereby excluding the most vulnerable children from the analysis. This could lead to an underestimation of the true burden of wasting in the population, as the prevalence figures would only reflect those who survived to be included in the study. To address this limitation, it is essential to acknowledge that the estimates provided in this study reflect the prevalence of wasting among infants who survived to the point of data collection. The exclusion of deceased infants who are typically those most severely affected by wasting may result in a healthier sample than what exists in the general population. In future studies, it would be beneficial to account for morbidity. Nonetheless, despite this potential underestimation, the findings from this study remain valuable in highlighting the prevalence and distribution of wasting among surviving infants, providing a useful indication of the current status of child malnutrition in the study area.**

Line 454 - Authors should also list strengths of their study.

**Corrected: Yes, the strength of the study added as follows. “The study used a comprehensive approach and robust dataset, which includes a large sample of infants, enhancing the reliability and generalizability of its findings on infant wasting. By examining a variety of socio-demographic, maternal, and infant-related factors such as maternal education, breastfeeding practices, and socio-economic status. Additionally, the study provides valuable insights into modifiable determinants of malnutrition and potential intervention points. Focusing on early-life factors, particularly feeding practices like exclusive breastfeeding, emphasizes a crucial developmental period where timely interventions can significantly impact infant health outcomes, contributing to the prevention of wasting and other forms of malnutrition.”**

Line 457 - ...Southern regions OF ETHIOPIA.

**Corrected: Yes, *changed to SNNPR.***

Other points
Line 465 - AcknowledgmentS not Acknowledgment

**Corrected: Yes, *Acknowledgments***

References
URLs or DOIs should be added to the following citations: 1, 3, 4, 5, 8, 13, 15, 23, 25, 27, 34, 38

**Corrected: Yes, URLs added for the references, 1, 3, 4, 5, 8, 13, 15, 23, 25, 27, 34, 38.**

**Reviewer #2**

Reviewer #2: It was a pleasure to review a paper on such an important global issue. I believe that this article is technically sound, and that the data provided supports the conclusions drawn by the researchers. From my experience with statistical analysis, I believe that the analytical protocols were relevant, rigorous, and justified in context. However, my skills in this area are not to a level where I can confidently endorse the statistical analysis here. This does not at all mean that the analysis was not rigorous and appropriate, as per the publication guidelines, but simply that I am not in a position to make this judgment. I believe the authors have made available all relevant and supporting data, including the raw endline survey data as an appendix and all tables of summarized data.

For point 4 I had selected 'no' because I have a few suggestions here. Overall, this paper was written clearly and in correct English. But in some places, I think some rewording would improve clarity:

Line 97: What is meant by the 'knee-bent' position?

**Corrected: Yes, definition is added at the introduction part. “*The knee-bent position refers to a*** ***natural posture where the infant's legs are not fully straight but rather flexed at the knees.”***

Line 99-101: The sentence beginning 'Moreover' could be rewritten, as it seems to change direction halfway through and is therefore a little unclear.

**Corrected:** **Yes, this sentences is corrected as follows, “*Additionally, calculating the WLZ using the WHO 2006 growth standards presents a challenge for infants with a length less than 45 cm, particularly for those born preterm or small-for-gestational-age.”***

Line 105-107: The sentence beginning 'Maternal' - should 'including' be 'included'?

**Corrected:** **Yes, *'including' changed to 'included'***
Line 187-189: Were these two sentences supposed to be just one sentence?

**Corrected:** **Yes, and it was corrected as follows. “*Infant feeding indicators are the proportion of infants aged 0-5 months who are fed exclusively on breast milk, with no other food or drink, including water, except for oral rehydration solutions (ORS) and drops or syrups containing vitamins, minerals, and medicine.”***

Lines 308 + 311: You have repeated data here.

**Corrected:** **Yes, *the repeated data removed.***
Line 422-424: This sentence could be reworded to improve clarity. Maybe, "This could be due to early initiation of breastfeeding have a protective effect against infections and reducing newborn mortality."?

**Corrected**: **Yes, corrected as follows “*This could be due to early initiation of breastfeeding have a protective effect against infections and reducing newborn mortality.”***

This note might be a little pedantic on my part, but I think replacing 'done' with 'completed' or 'conducted' reads better. (Lines 28, 112, and 422).
**Corrected: Yes, Done changed to 'conducted'**

In the results section on wasting under 6 months (beginning Line 321) the percentages presented switch between representing the proportion of wasting and the proportion without wasting. E.g., "Among mothers with no formal education, 66.7% of their infants were wasted" (Line 326) and "Regarding antenatal visits, 59.3% of infants who has four or more ANC visits were not wasted" (Line 329). I think it would be clearer to consistently report either the percentage of wasting or percentage without wasting. This might also help to avoid readers misinterpreting the data here.

**Corrected: Yes,** **this sentence was changed by describing the trends of wasted and non-wasted groups with the maternal educational level. “*A higher proportion of wasted children, 66.7% are from mothers with no formal education, indicating an association between lower maternal education and higher rates of child wasting. Among mothers with primary education, 31.2% of children are wasted, which is lower than those with no formal education. Only 2.1% of wasted children are from mothers with secondary or higher education, suggesting that higher maternal education is associated with a lower prevalence of child wasting.”***

Three other thoughts I had whilst reviewing this paper:

1. In the introduction (Line 113) it states that the policy and program implications of the findings will be discussed. However, these are not discussed in the paper, but rather mentioned in the conclusion as a future consideration. In my view, a 'discussion' of the policy and program implications would include some detail on what is currently in place followed by suggestions (based on the findings) of how these could be updated/improved to reduce wasting in infants in this area. I would recommend including some discussion or rewording the introduction to change "We also discuss" to "We also make recommendations for future policy and program directions" (or something along those lines).
**Corrected: Yes,** **corrected as follows “*We also make recommendations for future policy and program directions, focusing on improving maternal and child health outcomes*.”**

2. The survey options for number of ANC visits were "1-3 visits" or "4 or more visits". Upon review of the feasibility study from which this survey was taken, at least 1 ANC visit was an eligibility requirement for inclusion into this survey. Is it possible that there are women who have not attended any ANC visits in this area and were therefore not included in the survey? If so, this would be a limitation worth mentioning, as if a large number of women were excluded on this basis, these results may not accurately reflect the current state of infant wasting in this area. It is also possible that this number if very small, or that all women attended at least 1 ANC visit, but it is still something to consider in my opinion.

**Response: The study clusters were based on health center catchment areas, comprising 10 clusters across three woredas in the Somali region and 20 clusters across four woredas in the Southern regions, totaling 30 clusters using stratified randomization. Since the study focused on healthcare facilities, the exclusion of non-ANC attendees may not significantly affect the study's ability to assess the prevalence of infant wasting within the population that interacts with the healthcare system. In this context, the study's findings may more accurately reflect the outcomes of those with access to formal healthcare services. Furthermore, individuals who did not attend ANC may have different socio-economic or geographical barriers, and their absence from the study does not necessarily distort the overall findings related to healthcare access and maternal health.**

**The study clusters were defined as the health center catchment areas, which further strengthens the focus on those within a healthcare system's reach and ensures that the sample accurately represents the target population.**

3. The first thing discussed in the discussion is the prevalence of wasting in this study compared to other similar areas. Although this is important to note as it highlights the global scale of this issue, I do not think it is the first thing to be discussed here. Instead, I think discussing the findings of this study and highlighting the key factors found to be associated with wasting would be best, as these are the findings that directly address the research question. This could then be tied to a wider discussion of wasting in other geographical areas, providing potential explanations for differences in wasting prevalence (as you have done). In general, I think starting the discussion with study-specific findings and then broadening to the wider context is best.

**Response: Thank you for your thoughtful feedback. I appreciate your suggestion to begin the discussion with the study-specific findings before expanding to the broader context. However, I opted to start with the prevalence of wasting was intended to highlight the significance of the issue in this region, setting the stage for a deeper analysis of the associated factors. I felt this approach would provide essential context, helping to highlight the relevance of the findings in a broader, and global framework before delving into the specific results. I hope this clarifies my reasoning, but I value your perspective and will carefully consider your suggestion moving forward.**

Overall, this is a considered and well-written report on infant wasting and breastfeeding practices in Ethiopia. With some minor revision, I believe this paper will be a great contribution to the literature.
