# Supplementary material for: Association between wasting and inadequate breastfeeding practices among infants under six months in SNNPR and Somali regions of Ethiopia: A multilevel cross-sectional study
Source: PLoS One. 2025 Feb 7;20(2):e0318323. doi: 10.1371/journal.pone.0318323 (PMC11805366; doi:10.1371/journal.pone.0318323)
Supplement: Attachment — Submitted filename: Response to Reviewers. 2.docx [file pone.0318323.s006.docx]

**Summary of Response to Reviewers Comment**

**Based on the reviewers' comments, the following revisions were made:**

1. The first paragraph was revised as follows: “This study aims to investigate the relationship between breastfeeding practices and wasting among infants under six months of age in Ethiopia, addressing a critical gap in understanding wasting in this vulnerable age group.”
2. Throughout the manuscript, the term "Southern" was replaced with "SNNPR" for consistency.
3. In Tables 4 and 5, spaces were added between values and parentheses, and commas were used to improve clarity.
4. The level of precision for all numerical data in the manuscript, both in text and tables, was standardized and corrected.
5. For the comment raised in Line 348, the explanation is as follows, the percentages for wasted and non-wasted children were calculated independently within each age group. For wasted children, the percentages represent their distribution within the total number of wasted children across all age groups, summing to 100%. Similarly, for non-wasted children, the percentages represent their distribution within the total number of non-wasted children, also summing to 100%. These calculations were done separately for wasted and non-wasted categories, and the percentages are not combined into a single total. To address any potential confusion, the sentence in the manuscript was reworded for clarity.

**Reviewer Comments**

**Reviewer #1**

General Comments
ABSTRACT
Line 23 - Subsection is Background but main body of paper uses Introduction

**Corrected:** **Yes, corrected in the manuscript as follows, “*Introduction changed to Background”***

Line 27 - "...age in ^THE SNNPR..."

**Corrected:** **Yes, corrected as follows, “*in the SNNPR and Somali regions of Ethiopia”.***

Line 30 - Authors have used "Southern". Please use only "SNNPR" notation throughout for consistency.

**Corrected:** **Yes, corrected, *"Southern", changed in to "SNNPR".***

Line 32 - I would drop the line about STATA from the abstract. Irrelevant detail for an abstract.

**Corrected:** **Yes, removed the word written in line 32.**
Line 37 - Trailing zero missing from "1.5".

**Corrected: Yes, corrected by adding zero*, 1.5 to 1.50***
Line 38 - Inconsistent level of precision 1.52 then 1.0, 2.3. Please change to use a consistent number of decimal places.

**Corrected: Yes, corrected by adding the numbers as follows,** 1.52; CI:1.00, 2.30.
Line 39 - Trailing zero mnissing from "1.5"

**Corrected: Yes, corrected by adding zero*, 1.5 to 1.50***

Line 40 - Missing trailing zeroes on 0.4 and 0.3

**Corrected: Yes, corrected by adding zero*, “0.40 and 0.30”***

Line 41 - Suggest adding some words to clarify if "...independently associated with wasting" mean those variables were significant during multivariable regression or just in bivariable. I presume the authors mean the multivariate regression but please make this clearer.

**Corrected: Yes, corrected as follows, *“were independently associated with wasting in the multivariable analysis”.***

Line 42 - Subsection is Conclusion but main body of paper uses Discussion

***Thank you for your valuable feedback. We acknowledge the inconsistency in terminology between the "Conclusion" subsection and the use of "Discussion" in the main body of the paper. In preparing the abstract, we followed the PLOS ONE format, which is unstructured and typically includes only the background, methods, results, and conclusions. This format is widely accepted and ensures clarity, conciseness, and alignment with PLOS ONE's submission guidelines***.
Line 45 - "may help" rather than "can help". This study was not an intervention so cannot make that claim, only suggest.

**Corrected: Yes, corrected by changing in to *"may help".***


\# INTRODUCTION
Line 65 - First paragraph should include a brief description of the study aim. Authors have reworded the final paragraph of the Introduction but have not added this to the first paragraph, as requested in the previous review. Having to read multiple pages of introduction without knowing the purpose of the study is frustrating.

**Corrected: Yes, the first paragraph was revised to include the aim of the study at the end, as follows: “*This study aims to investigate the relationship between breastfeeding practice and wasting among infant under six months of age in Ethiopia, addressing a critical gap in understanding wasting in this vulnerable age group.”***

Line 75 - "deviation" should be plurarlised to "deviations"

**Corrected: Yes, corrected by adding “s”, *"deviations"***

Line 94 - Were the 9.4% of children with wasting at the national level or within the regions of interest?

**Corrected: Yes, corrected as follows, “*9.4% of infants under six months of age were identified as wasted at the national level.”***

Line 102 - This should be changed back to "Additionally, the mid-upper arm circumference IS not typically for this age group."

**Corrected: Yes, corrected as follows, “*Additionally, the mid-upper arm circumference is not typically for this age group.”***

Line 107 - I don't think this should be a new paragraph.

**Corrected: Yes, corrected**

Line 122 - Consider mentioning that end-line data were used, since it was taken from an intervention study.

**Corrected: Yes, corrected as follows “*This analysis was conducted using the end-line household survey data from a maternal nutrition intervention study.”***

Line 145 - Replace with Southern with SNNPR for consistency.

**Corrected:** **Yes, corrected, *"Southern", changed in to "SNNPR".***

Line 148 - Does Southern mean SNNPR?

**Corrected:** **Yes, corrected, *"Southern", changed in to "SNNPR".***

Line 156 - Does Southern mean SNNPR?

**Corrected:** **Yes, corrected, *"Southern", changed in to "SNNPR".***

\# METHODS
Line 190 - "The ^PRIMARY study outcome..." since mutiple outcomes are discussed.

**Corrected:** **Yes, corrected by adding, *“The primary study outcome variable was wasting among infants under six months of age”***

Line 207 - I don't think a new sentence is needed at "The age of the infant...". If so, then perceived birth size should also be a new sentence.

**Corrected:** **Yes, corrected.**

Line 210 - "Infant feeding indicators WERE..." Consistence use of tense.

**Corrected:** **Yes, corrected by changing "are" to "were."**

Line 214 - Suggest "The following indicators were calculated..."

**Corrected:** **Yes, corrected by adding “*the following indicators were”***

Line 214 - Inconsistent use of capitalisation in this paragraph. Previous paragraphs do not capitalise items in the list. Suggest that "Infant ever...", "Timing of..." etc do not need capital letters.

**Corrected:** **Yes, corrected as follows, “*infant ever breastfeeds; timing of breastfeeding initiation; colostrum feeding; pre-lacteal feeding; bottle feeding; started cow/goat/camel milk; started solid, semi-solid, and soft food were coded as "no" and "yes."***

Line 225 - As above, inconsistent use of capital letters and list make this difficult to follow for the reader.

**Corrected:** **Yes, revised by changing the capital letters to lowercase.**

Line 231 - Missing period at the end of sentence.

**Corrected:** **Yes, revised by adding a period at the end of the sentence.**

Line 232 - Citation for wealth index should be given in this sentence, rather than the end of **paragraph.**

**Corrected: Yes, the citation for wealth index was given.**

Line 246 - "Descriptive statistics WERE..."

**Corrected:** **Yes, revised by changing "was" to "were."**

Line 249 - Suggest removing extended explanation of 95% CI's "...providing a range within which 250 the true population parameters are expected to lie with 95% confidence, adding 251 insight into the precision and reliability of our estimates."

**Corrected:** **Yes, corrected as follows,** ***The prevalence of wasting was determined using proportions with 95% confidence intervals (CIs), to indicate the precision of the estimates.***

Line 259 - "...significance WAS declared..."
**Corrected: Yes, revised by changing "were" to "was."**

\# RESULTS
Line 295 - Parentheses around 67% and 32.8% would be appropriate.

**Corrected:** **Yes, corrected by adding parentheses, (67.2%) and (32.8%)**

Line 296 - Inconsistent precision 67% and 32.8% use different number of decimal places.

**Corrected:** **Yes, corrected as follows,** **(67.2%) and (32.8%)**

Line 296 - Parentheses around 63.% and 36.9% would be appropriate.

**Corrected:** **Yes, corrected by adding parentheses,** **(63.1%) and (36.9%).**

Line 297 - Inconsistent precision 63% and 36.9% use different number of decimal places.

**Corrected:** **Yes, corrected as follows, “(63.1%) and (36.9%).”**

Line 304 - Add trailing zeroes where needed. For example, "Middle" should be 20.0, not 20. Please check all rows for this issue.

**Corrected:** **Yes, the numbers in Table 1 were updated as follows: "Middle, Rich, and Richer" were changed to 20.0.**

Line 308 - Inconsistent precision 42% has fewer decimal places compared to rest of paragraph.

**Corrected: Yes, revised by changing "42%" to "42.1%"**

Line 319 - Add trailing zeroes where needed. For example, "3-6 month" should be 61.0, not 61. Please check all rows for this issue.

**Corrected:** **Yes, revised by changing the numbers in Table 2, “61 to 61.0”.**

Line 330 - "Exclusive breastfeeding status..." should be in the Methods section?

**Corrected:** **Yes, revised by removing the sentence, as the "Exclusive breastfeeding status" is already discussed in the methods section.**
Line 335 - Add trailing zeroes where needed. For example, "Stunted Yes" should be 10.0, not 10. Please check all rows for this issue.

**Corrected: Yes, revised by changing "10" to "10.0"**
Line 340 - Inconsistent precision 58% has fewer decimal places than 41.8%.

**Corrected: Yes, revised by changing "58%" to "58.2%"**

Line 342 - Change tense throughout "...wasting WAS higher at FIVE months..." rather than IS.

**Corrected:** **Yes, corrected as follows, *“******The proportion of wasted children varies with age, wasting was higher at five months, where 26.2% of wasted cases are observed. Wasting was lowest at one months, 8.5% and shows an increase at two months, 20.6% remaining relatively high from two to five months.***

Line 342 - Numbers of nine or less should be expressed as words. "...wasting WAS higher at five months...". Change throughout paragraph.

**Corrected:** **Yes, corrected as follows, *“The proportion of wasted children varies with age, wasting was higher at five months, where 26.2% of wasted cases are observed. Wasting was lowest at one months, 8.5% and shows an increase at two months, 20.6% remaining relatively high from two to five months.”***

Line 348 - It is unclear what other category there may be excepted wasted or not wasted. If 26.2% of children are wasted at five months and 24.7% of children are not wasted at five months, what has happened to the remaining ~50%?

**Corrected:** **Yes, discussed in the summary of the response to the reviewer, *the percentages for wasted and non-wasted children were calculated independently within each age group. For wasted children, the percentages represent their distribution within the total number of wasted children across all age groups, summing to 100%. Similarly, for non-wasted children, the percentages represent their distribution within the total number of non-wasted children, also summing to 100%. These calculations were done separately for wasted and non-wasted categories, and the percentages are not combined into a single total. To address any potential confusion, the sentence in the manuscript was reworded for clarity.***

Line 356 - Parentheses around 66.7% would be appropriate.

**Corrected:** **Yes, corrected by adding parentheses.**

Line 360 - Remove "suggesting that higher maternal education is associated with a lower prevalence of child wasting." This is a point for Discussion, not Results.

**Corrected: Yes, revised by removing the sentence**

Line 374 - Missing trailing zeroes in some rows. For eample, Prevalence of "Secondary and above education" is given as "5(1,13.9)" but should be given as "5.0 (1.0, 13.9)".

**Corrected: Yes, revised by adding trailing zeroes in Table 4, for example, "5(1,13.9)" was changed to “5.0 (1.0, 13.9)”.**

Line 374 - Change column header to "Prevalence (95% CI)" with parentheses

**Corrected:** **Yes, corrected as follows*, "Prevalence (95% CI)".***

Line 374 - Spaces should be placed between values and parentheses to make this table easier to read. For example, "20-24" in the Prevalence column - 14.2(9.3,20.3) should be 14.2 (9.3, 20.3). All of these values in the table should be formatted as such for clarity.

**Corrected: Yes, revised by adding spaces between values and parentheses in Table 4.**

Line 395 - As above. Please add spaces to make values more easily readable throughout this table.

**Corrected: Yes, corrected by adding space between values and parentheses**

Line 395 - Hyphens used for 95% CI in this table but commas have been used in Table 4. Please go through the manuscript and choose one notation and use consistently.

**Corrected: Yes, revised by using commas consistently in both Table 4 and Table 5.**

Line 395 - Suggest "Child age (months)" to make it clear it is measured in months.

**Corrected:** **Yes, corrected by adding, “Child age (completed months)”**

\# DISCUSSION
Line 404 - "Socio-demographic" does not need a capital letter and no capital is used on Line 584 for the same phrase.

**Corrected:** **Yes, corrected.**

Line 404 - Add "... in the Somali and SNNPR regions of Ethopia"

**Corrected: Yes, corrected by adding, in the Somali and SNNPR regions of Ethiopia"**

Line 411 - I appreciate the addition of the reference to the regional statistics as per my suggestion of my previous review. But as the authors point out, this talks about a different age group. I was wrong to suggest this comparison. I leave it to the authors to decide if they wish to remove this comparison.

**Corrected: Yes, revised by removing the sentence that refers to a different age group.**

Line 418 - Inconsistent precision 14.2% and 16% use different numbers of decimal places.

**Corrected: Yes, revised by adding a trailing zero, “16.0%”.**

Line 423 - As per Line 418 comment.

**Corrected:** **Yes, corrected as follows, “*72.2%***”

Line 445 - Clarify if 1.48 or 1.50.

**Corrected: Yes, corrected. The prevalence of the odds ratio was 1.50, the value 1.48 was a typographic error.**

Line 448 - What rate did Negatu et al observe, specifically? Same for studies in Nigeria and Bangladesh. Please report the ratios from these studies here.

**Correction: Yes, corrected by including the prevalence of the odds ratio for the studies by Negatu et al. and Bangladesh. However, for the study conducted in Nigeria, the prevalence was not clearly specified.**

Line 473 - As above, please report the specific ratios found in citations number 44 and 45 directly in the Discussion.

**Corrected:** **Yes, corrected as follows, “*This study found that infants who did not initiate breastfeeding within one hour of birth had 52% higher odds of wasting compared to those who started breastfeeding within the first hour. This finding aligns with research conducted across 20 developing countries using demographic health survey data, which reported a 31% increase in the odds of wasting among infants who delayed breastfeeding initiation [40]. Similarly, a study from South Asia demonstrated that early breastfeeding initiation reduced the odds of wasting by 8% [41]. These findings highlight the importance of timely breastfeeding initiation in reducing the risk of wasting across various populations.”***

Line 476 - "...HAVING a protective..."

**Corrected:** **Yes, corrected as follows, “*This could be due to early initiation of breastfeeding having a protective effect against infections and reducing newborn mortality.”***

Line 508 - What ratio did citation 19 find for sex differences? Please report directly here so the reader can see if it compares to the authors' value of 1.50.

**Corrected:** **Yes, corrected as follows, “*This study showed that males were 50% more likely to be wasted than females. This finding aligns with data from the 2019 Ethiopian Mini Demographic Health Survey, which reported that being female was associated with 30% lower odds of wasting [15]. Similarly, a study conducted in three disadvantaged East African Districts, Rwanda, Uganda, and Tanzania among children under five years of age found that being female reduced the odds of wasting by 14% [47].”***
Line 510 - As above for citation 51.

**Corrected:** **Yes, corrected.**

Line 581 - "StrengthS" rather than "Strength"

**Corrected: Yes, revised by adding "s", "Strengths".**

Line 592 - "LimitationS" rather than "Limitation"

**Corrected: Yes, corrected by adding "s", "Limitation"**

Line 592 - Add a brief note to explain that information about singleton/multiple births was not collected.

**Corrected:** **Yes, corrected by adding no information were collected about singleton/multiple births in the limitation section.**

Line 602 - Please use either SNNPR or Southern throughout the paper (Ctrl + F!)

**Corrected:** **Yes, corrected by changing Southern to SNNPR.**
