# Supplementary material for: Association between wasting and inadequate breastfeeding practices among infants under six months in SNNPR and Somali regions of Ethiopia: A multilevel cross-sectional study
Source: PLoS One. 2025 Feb 7;20(2):e0318323. doi: 10.1371/journal.pone.0318323 (PMC11805366; doi:10.1371/journal.pone.0318323)
Supplement: Attachment — Submitted filename: Response to Reviewers 3.docx [file pone.0318323.s007.docx]

**Response to Reviewers Comment**

Thank you for your valuable comments and feedback. Based on your suggestions, the following revisions have been made:

**# Abstract**
All changes implemented from previous review. The abstract is now more consistent and has better grammar/readability.

**# Introduction**
Purpose of study being included in the first paragraph is a significant improvement, thank you.

Line 99 - "...used..." has been deleted but I think a word like "used" was meant to replace it? You could probably just delete this sentence since it distracts the reader, if you want.

**Corrected:** **Yes, corrected by removing the sentence.**

**# Methods**
It looks like all grammar and punctuation issues have been fixed. The section is now easier to read. Thanks!

**# Results**
Line 280 - Minor point: sentence begins with a numeral. This is not typical. Consider minor rewrite. (Contrast to Line 298.)

**Corrected:** **Yes, corrected as follows “More than half of the mothers (68.0%) were housewives, and 52.3% had no formal education.”**

Line 282 - "while the REMAINDER (32.8%)..." rather than REMAINING

**Corrected:** **Yes, corrected by changing remaining to reminder.**

Line 324 - Suggestion for improved sentence and addition of a missing percentage "Wasting was lowest at age one month (8.5%), increased by age two months (20.6%) and remained relative high from ages two to five months (XX.X%)"

**Corrected: Yes, corrected as follows, “The age distribution of children in the wasted group was 11.4% aged zero months, 8.5% aged one month, 20.6% aged two months, and 59.5% aged three to five months. The age distribution of children in the non-wasted group was 9.3% aged zero months, 15.6% aged one month, 20.0% aged two months, and 55.1% aged three to five months."**

Line 327 - As per the authors' response to the previous review, this paragraph is meant to describe the age DISTRIBUTION of the wasted children, and, separately the age DISTRIBUTION of the non-wasted children so the percentages in the waste group sum to 100% and the percentages in the non-wasted group sum to 100%. Please rewrite this paragraph in a more systematic way. I suggest the following (but with the correct numbers!) "The age distribution of children in the wasted group was 10% age zero months, 20% age one month, 50% aged two to five months and 20% aged over five months. The age distribution of children in the non-wasted group was 30% age zero months, 10% age one month, 20% aged two to five months, and 40% aged over five months."

**Corrected: Yes, corrected as follows,** **" The age distribution of children in the wasted group was 11.4% aged zero months, 8.5% aged one month, 20.6% aged two months, and 59.5% aged three to five months. The age distribution of children in the non-wasted group was 9.3% aged zero months, 15.6% aged one month, 20.0% aged two months, and 55.1% aged three to five months."**

Line 333 - "A HIGH proportion..."

**Corrected: Yes, corrected by changing higher to high.**

Line 337 - "...WERE from mothers"

**Corrected: Yes, corrected by changing “are to where”.**

Line 346 - Some spaces have been added but not in all columns. The Wasting Yes/No columns should also have the formatting corrected from, e.g., 55(7.7) to 55 (7.7)

**Corrected: Yes, space was added to the Wasting Yes/No columns.**
Line 346 - If you are reporting confidence intervals you do not need to report p-values at the same time. The overlap of the confidence intervals demonstrates that, e.g., prevalence of wasting did not vary significantly for any age compared to your reference group (15-19 years).

**Corrected: Yes, the p-values reported were removed.**

Line 357 - "Wasting among infants who were not exclusively breastfed was 1.50 times more likely compared to infants who were exclusively breastfed (APR = 1.50; 95% CI:1.02, 2.21)." Deleting some words to make the sentence clearer.

**Corrected: Yes, "Infants who were not exclusively breastfed were 1.50 times more likely to experience wasting compared to those who were exclusively breastfed (APR = 1.50; 95% CI: 1.02–2.21)."**

Line 360 - Suggest using exact same phrasing as previous sentence, instead of switching to "52%". "Wasting among infants who initiated breastfeeding more than one hour after birth were 1.52 times more likely compared to infants who start breasteeding less than one hour after birth (APR = 1.52; 95% CI: 1.00, 2.30)."

**Corrected: Yes, corrected as follows, “Wasting among infants who initiated breastfeeding more than one hour after birth were 1.52 times more likely compared to infants who started breastfeeding less than one hour after birth (APR = 1.52; 95% CI: 1.00, 2.30)."**

Consistent levels of precision in tables and text have improved the flow of the writing. Thanks!
 **\# Discussion**
Line 384 - "...Barisal district (18.8%)..." - Remove the word "at" before the (18.8%).

**Corrected: Yes, by removing “at”**

Line 433 - Reference needed to support this claim about gastrointestinal diseases. May already be supported by Ref 39 in the next sentence?

**Yes, the sentence was supported by reference 39.**
